# Effects of age on psychosocial working conditions and future labour market marginalisation: a cohort study of 56,867 Swedish twins

**DOI:** 10.1007/s00420-021-01704-z

**Published:** 2021-05-07

**Authors:** Mo Wang, Pia Svedberg, Jurgita Narusyte, Kristin Farrants, Annina Ropponen

**Affiliations:** 1 Division of Insurance Medicine, Department of Clinical Neuroscience, Karolinska Institutet, 171 77 Stockholm, Sweden; 2 Finnish Institute of Occupational Health, Helsinki, Finland

**Keywords:** Job demands, Job control, Social support, Sick leave, Disability pension, Unemployment

## Abstract

**Purpose:**

Previous studies have reported an elevated risk of sickness absence (SA) and disability pension (DP) due to adverse psychosocial working conditions, yet the influence of age and familial factors on the associations have not been examined. We aimed to investigate associations between psychosocial working conditions and labour market marginalisation (LMM) in terms of unemployment, SA and DP adjusting for familial confounding and possible differences in these associations with different age groups and different unemployment and sick leave days.

**Methods:**

All twins living in Sweden in 2001, aged 16–64 years and not on old-age pension or DP were included (*n* = 56,867). The twins were followed from 2002 to 2016 regarding unemployment, SA and DP. Cox proportional hazards regression models were performed for the whole sample, and for discordant twin pairs, in five age groups.

**Results:**

Each one-unit increase in job demands and job control was associated with a lower risk of unemployment, SA and DP in all age groups. Moreover, each one-unit increase in social support was associated with an increased risk of 1–30 days unemployment in individuals older than 45 years and SA and DP. Social support decreased the risk of unemployment longer than 365 days in age groups 16–25 and 36–45 years. In the discordant twin pair analyses, the estimates attenuated towards statistical non-significance.

**Conclusion:**

Even though familial factors seem to influence the associations between psychosocial working conditions and LMM, improving psychosocial working conditions by for example promoting high job control and social support at workplace may reduce the risk of future short- and long-term LMM in all age groups.

**Supplementary Information:**

The online version contains supplementary material available at 10.1007/s00420-021-01704-z.

## Introduction

Work is considered to be crucial as it provides financial support, a structure for daily routine, and is a source of identity and social status (Saunders 2014). When these advantages are interrupted by unemployment or work incapacity including sickness absence (SA) and disability pension (DP), poorer physical and mental health and higher rates of mortality might be presented (Saunders 2014). In many OECD countries, labour market marginalisation (LMM) measured by unemployment and work incapacity has been deemed as a serious societal problem, affecting both public health and economic development (OECD 2010). For instance, when people cannot independently support themselves through paid employment, there may be enormous challenges for societies, as the costs for loss of work productivity and welfare benefits will grow considerably. However, several previous studies only included unemployment when they measured LMM. The recent studies suggest conceptualising LMM from a social insurance perspective and including measures both based on the medical assessments (work incapacity in terms of SA and DP) and measures not based on medical assessments (unemployment) (Helgesson 2018), otherwise there is a risk to underestimate the true risk and consequences of LMM. In the recent decades, the incidences of SA and DP have increased in many Western countries (Social insurance in figures 2016), which may limit possibilities for labour market participation. Therefore, it is of high importance to identify work-related risk factors for LMM to achieve a sustainable working life.

The Job Demand–Control–Support model developed by Karasek and Theorell (Karasek 1979), has extensively been used to measure psychosocial working conditions for understanding and interpreting the relationships between the characteristics of health, work and well-being (Kivimaki 2010; Stansfeld 2006). Most of the studies of health results have mainly applied the Job Demand–Control model with job strain which is defined as the situation where one experiences high job demands combined with low control at work. However, there is a need for more knowledge regarding the separate effects from job demands, job control and social support on subsequent LMM. A number of studies have reported the associations between adverse psychosocial working conditions and increased risk of overall SA and DP as well as SA and DP due to mental and musculoskeletal diagnoses (Mather 2015; Samuelsson 2013; Norberg 2020; Ropponen 2013; Canivet 2013; Lahelma 2012; Clumeck 2009). Two recent published studies examined individuals with paid work in Sweden and found that women in occupations with low job demands and low job control, and men in occupations with high job demands and low job control, had a higher risk of long-term SA and DP (Norberg 2020; Farrants, et al. 2020). Two prospective cohort studies reported that high job demands and job strain were associated with an elevated risk of SA due the mental disorders (Mather 2015; Clumeck 2009). Furthermore, the risk of DP due to mental and musculoskeletal disorders was observed to be associated with low job demands and job control (Samuelsson 2013; Ropponen 2013). However, the knowledge regarding associations between psychosocial working conditions and unemployment is sparse. Norberg et al. (2020) found that unemployment was predicted by low job demands and job control. Furthermore, not much is known about risk of number of days of unemployment or SA in relation to psychosocial working conditions. Such knowledge is needed to develop different approaches for preventing short- and long-term LMM.

Another knowledge gap in this area includes impact of age in the associations. As influence from psychosocial working conditions in work is evident throughout the whole working life, it is critical to investigate associations between psychosocial working conditions and LMM from a life course perspective. A number of previous research suggest an increased risk of SA and DP among older individuals (Allebeck et al. 2004; Karlsson 2006) while there has been an increase in unemployment and SA/DP due to mental disorders among young adults in several developed countries (Social insurance in figures 2016; OECD 2015). In addition, a Danish study showed that psychosocial working conditions were associated with long-term SA and DP among older workers (Sundstrup 2018). This study will fill this knowledge gap by examining the associations utilising 10-year age groups across the life course.

Moreover, the vast majority of studies exploring the contribution of psychosocial work conditions to the risk of unemployment, SA and DP has disregarded the potential influence of familial factors. This can be investigated by studying discordant twin pairs, who are optimally matched on genetics (100% for monozygotic (MZ) and on average 50% for dizygotic (DZ) twin pairs) and common rearing environment (100% for both MZ and DZ twin pairs when reared together), referred to as familial factors as well as age and sex (for the same-sexed pairs) (Carlin 2005). It is reasonable to assume that familial factors may influence the associations since twin studies have documented that SA has a moderate genetic component, explaining 36–50% of the total variance, among both women and men (Svedberg 2012; Gjerde 2013; Seglem 2019), as does DP (Gjerde 2013; Narusyte 2011; Harkonmaki 2008). By comparing the results from the whole cohort, the influence of familial factors can be seen if the analyses of discordant twin pairs are substantially different. In contrast, if the results from analyses of discordant twin pairs are similar to the associations of the whole cohort, then factors unique to each individual are expected to be more crucial (i.e., indicating less or non-existing effect of familial factors). In this study, we included a large sample of twins of working age while using population-based register data.

## Aims

The aims of the study were to investigate (1) associations between psychosocial working conditions and future LMM, measured as unemployment, SA and DP while accounting for familial confounding (genetics and shared environment) and (2) possible differences in these associations with regard to age and unemployment and sick-leave days.

## Methods

### Study population and data sources

**Fig. 1:**
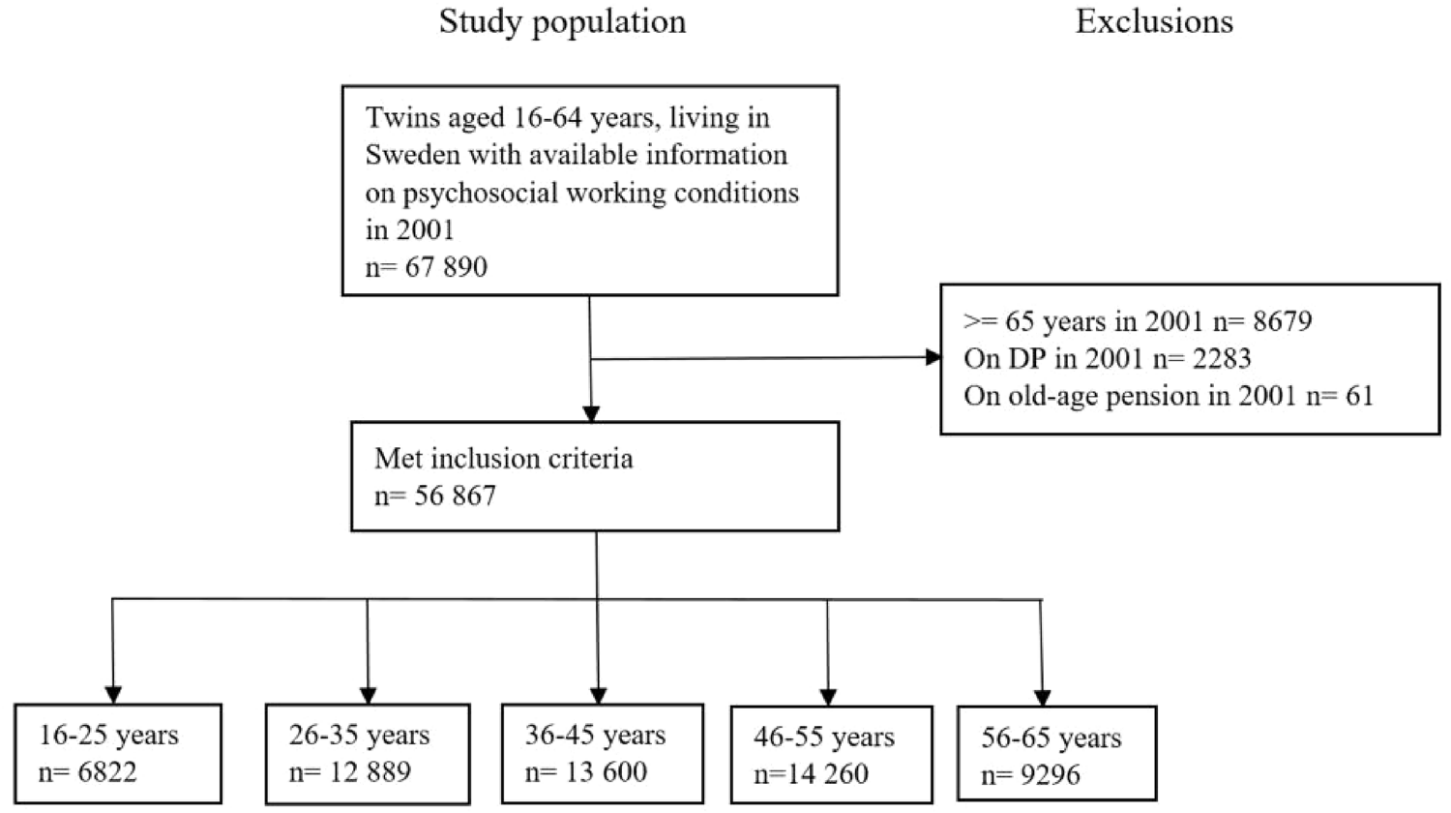
Flow chart for the study population

This prospective twin cohort study was based on data from the Swedish Twin project of Disability pension and Sickness absence (STODS) which includes all twins from the Swedish Twin Registry (STR) born 1925–1990 in Sweden, i.e., 119,907 twin individuals (Magnusson 2013). We included individuals who were registered as living in Sweden, aged between 16 and 64, and had available information on psychosocial working conditions in December 2001. Individuals who were older than 65 years, on DP or old-age pensioned in 2001 were excluded. This left a final sample of 56,867 twins. The sample included 20,690 complete twin pairs whereof 5406 MZ, 6098 DZ same sex, 2451 of unknown zygosity and 6735 opposite sex twin pairs. The study sample also included 15,487 twin individuals without their co-twin. The sample was further stratified into five age groups according to their age in 2001: 16–25 years, 26–35 years, 36–45 years, 46–55 years and 56–64 years (Fig. 1).

Information on exposure, covariates and outcomes was obtained from national registers by linking the unique personal identification number which are provided to participants (Ludvigsson 2009):

- From the Swedish Social Insurance Agency: MicroData for Analyses of Social insurance (MiDAS) register that includes dates for all SA (> 2 weeks) and DP spells from 1994 and onwards.
- From Statistics Sweden: the Longitudinal Integration Database for Health Insurance and Labour Market Studies Register (LISA) that contains information on psychosocial working conditions, unemployment, emigration and socio-demographic variables from 1990 and onwards (Ludvigsson 2019).
- From the National Board of Health and Welfare: The causes of death register that contains dates for all deaths from 1961 and onwards.

### Outcomes

The cohort was followed from 1st January 2002 until 31st of December 2016 with regard to the following measures of LMM: (Saunders 2014) unemployment; (OECD 2010) SA; (Helgesson 2018) DP using the date of the first such event. As there was no information on unemployment date in LISA, 1st January of the first calendar year with unemployment benefit was applied. Furthermore, the number of unemployment days during the follow-up was grouped in 3 categories: 1–30, 31–365 and more than 365 days. The number of sick-leave days during the follow-up was grouped in 2 categories: 1–30 and 31–365 days. SA more than 365 days were included as DP.

### Exposure

Information from the LISA register data in 2001 was used to assess psychosocial working conditions based on the Swedish psychosocial Job Exposure Matrix (JEM), developed by Fredlund et al. (2000). Based on the JEM, each Nordic Job Classification (NYK) occupation was assigned an age- and sex-specific mean score (range 1–10) for job demands, job control and social support. The scores for job demand, job control and social support were used as continuous variables in the statistical analyses. For each of the dimensions, higher scores indicated more optimal characteristics (low demands, high control and high support), while lower scores meant the opposite.

In the JEM, occupational codes were coded according to NYK while the occupational codes of the study population were coded according to Swedish Standard for Occupational Classification (SSYK by Swedish acronym). Therefore, we translated SSYK occupational codes into NYK. In NYK, there are 320 occupational 3-digit codes, covering different sector-based occupational groups, namely: technology, science, social science and art; healthcare and social work; administration and management; commercial work; agriculture forestry and fishing; transport; production and mining; and service and military work. For a more detailed description of translation of occupational codes, see Norberg et al. (2020).

### Covariates

Covariates included sex [women versus men (reference group)], levels of education (i.e., highest level of completed education, classified into elementary (0–9 years of education, reference group), medium (> 9–12 years in education) and higher education [> 12 years in education)], marital status [married (reference group) versus unmarried (including single, divorced and widow/widower)], having children living at home [no children living at home versus at least one child living at home (reference group)], type of living area [urban/semi-urban areas (including Stockholm, Göteborg and Malmö and cities with more than 90,000 inhabitants within 30 km distance from the centre of the city; reference group) versus semirural/rural areas (including small cities/villages)] and SA pyes versus no (reference group)] in 2001 (Table 1).

**Table 1:** Frequencies of socio-demographic factors, psychosocial working conditions, unemployment, sickness absence, disability pension and labour market marginalisation among 56,867 twins, stratified on age groups

	Whole sample	Age groups				
		16–25 years	26–35 years	36–45 years	46–55 years	56–64 years
	*n* (%)	*n* (%)	*n* (%)	*n* (%)	*n* (%)	*n* (%)
*N*	56,867 (100)	6822 (100)	12,889 (100)	13,600 (100)	14,260 (100)	9296 (100)
*Sex*						
Men	28,208 (49.6)	3238 (47.5)	6642 (51.3)	6873 (50.5)	7065 (49.5)	4390 (47.2)
Women	28,659 (50.4)	3584 (52.5)	6247 (48.5)	6727 (49.5)	7195 (50.5)	4906 (52.8)
*Zygosity*						
Monozygotic	13,864 (24.4)	2056 (30.1)	3475 (27.0)	2855 (21.0)	3298 (23.1)	2180 (23.5)
Dizygotic same sex	16,511 (29.0)	1427 (20.9)	2936 (22.8)	3939 (29.0)	4995 (35.0)	3214 (34.6)
Dizygotic opposite sex	19,290 (33.9)	1913 (28.0)	3868 (30.0)	4886 (35.9)	5268 (36.9)	3355 (36.1)
Unknown zygosity	7202 (12.7)	1426 (20.9)	2610 (20.3)	1920 (14.1)	699 (4.9)	547 (5.9)
*Education*						
Elementary (0–9 years)	10,731 (17.9)	975 (14.3)	870 (6.8)	1737 (12.8)	3345 (23.5)	3246 (34.9)
Secondary (> 9–12 years)	30,682 (54.0)	4567 (67.0)	7587 (58.9)	7715 (56.7)	6871 (48.2)	3942 (42.4)
Higher education (> 12 years)	16,012 (28.2)	1280 (18.8)	4432 (34.4)	4148 (30.5)	4044 (28.4)	2108 (22.7)
*Marital status*						
Married	24,522 (43.1)	132 (1.9)	3161 (24.5)	6491 (47.7)	8664 (60.8)	6074 (65.3)
Unmarried	32,345 (56.9)	6690 (98.1)	9728 (75.5)	7109 (52.3)	5596 (39.2)	3222 (34.7)
*Children living at home*						
Yes	28,530 (50.2)	3211 (47.1)	6261 (48.6)	9936 (73.1)	7395 (51.9)	1727 (18.6)
No	28,337 (49.8)	3611 (52.9)	6628 (51.4)	3664 (26.9)	6865 (48.1)	7569 (81.4)
*Type of living area*^a^						
Urban/semi-urban	39,914 (70.2)	5056 (74.1)	9519 (73.9)	9486 (69.8)	9490 (66.6)	6363 (68.5)
Rural	16,953 (29.8)	1766 (25.9)	3370 (26.2)	4114 (30.3)	4770 (33.5)	2933 (31.6)
*Psychosocial working conditions (Mean, SD)*						
Job demands (range 1–10, high score is high)	4.86 (0.70)	4.60 (0.61)	4.91 (0.71)	4.94 (0.71)	4.87 (0.71)	4.82 (0.71)
Job control (range 1–10, high score is high)	6.40 (1.28)	5.87 (1.25)	6.47 (1.27)	6.56 (1.22)	6.46 (1.28)	6.38 (1.28)
Social support (range 1–10, high score is high)	6.51 (0.62)	6.92 (0.49)	6.61 (0.56)	6.46 (0.58)	6.37 (0.63)	6.38 (0.64)
*LMM (2002–2016)*						
Unemployment 1–30 days	2037 (3.6)	387 (5.7)	585 (4.5)	495 (3.6)	406 (2.9)	164 (1.8)
Unemployment 31–365 days	8627 (15.2)	2153 (31.6)	2557 (19.8)	1921 (14.1)	1420 (10.0)	576 (6.2)
Unemployment > 365 days	6454 (11.4)	1006 (14.8)	1665 (12.9)	1534 (11.3)	1604 (11.3)	645 (6.9)
Sickness absence 1–30 days	12,164 (21.4)	1589 (23.3)	2986 (23.2)	3138 (23.1)	3239 (22.7)	1212 (13.0)
Sickness absence 31–365 days	15,619 (27.5)	1823 (26.7)	3502 (27.2)	4012 (29.5)	4415 (31.2)	1831 (19.7)
Disability pension^b^	5979 (10.5)	249 (3.7)	770 (6.0)	1334 (9.8)	2156 (15.1)	1470 (15.8)
Combined LMM^c^	37,653 (66.2)	5047 (74.0)	8733 (67.8)	9141 (67.2)	9992 (70.1)	4740 (51.0)

^a^Type of living area: Urban/semi-urban areas: Stockholm, Göteborg and Malmö and cities with more than 90,000 inhabitants within 30 km distance from the centre of the city; Semirural/rural areas: small cities/villages

^b^Including disability pension and sickness absence > 365 days

^c^LMM: Labour market marginalisation, measured by unemployment, sickness absence and disability pension

### Social insurance system in Sweden

Generally, SA benefits are granted by the Social Insurance Agency to all individuals in Sweden from the age of 16 years who have an income from work or unemployment benefits and who have a reduced work capacity due to a disease or injury (Social insurance in figures 2016). Individuals with a permanently impaired work capacity due to a disease or injury are eligible for DP benefits. Employers pay sick pay during the first 14 days. There is one qualifying day (more for self-employed) without benefits. A physician’s certificate is required after 7 days of self-certification. SA amounts to 80% of lost income while DP to approximately 65%. Entitlement to unemployment benefits presumes a defined minimum income from work.

### Statistical analyses

First, Cox regression models with competing risks were applied for the whole sample (*n* = 56,867). The proportional hazards assumption was tested by examining the log–log curves, which showed parallel curves in the tests. By using a Cox regression model, we could take care of competing events, that is, an outcome that prevents the occurrence of another outcome, especially important with regard to DP, where persons on DP no longer are at risk of unemployment and SA. To elucidate differences between age groups, the analyses were stratified by the five age groups. The individuals were followed until the outcome events or until the competing events; namely, death, emigration, old-age pension, age 65 and DP (in analyses with regard to unemployment and SA), whichever came first during the follow-up time in the model. Cox regression models were performed separately with each outcome as well as with a combined measure of LMM. The covariates were adjusted for in the multivariate models.

Second, a co-twin control analysis was carried out, using conditional Cox regression analysis. The analysis was conducted for MZ and same-sex DZ twin pairs discordant for the outcomes, i.e., one twin in a pair with unemployment, SA or DP and one twin without, all together and stratified by zygosity. In discordant twin-pair analyses, twins in a pair are optimally matched on genetic (100% for MZ pairs and on average 50% for DZ pairs) and shared environmental factors when reared together. Consequently, sex, age and familial factors are adjusted by matching in the conditional analysis and it is possible to assess the impact of familial confounding on the associations by comparing the results to the estimates of the whole sample, adjusted for sex and other covariates. In the stratified analyses on zygosity, there is a closer match in MZ twins when compared with DZ twins. Hence, if an association from the whole cohort was found to be weaker, but still remained among discordant DZ twin pairs, and is to a larger extent attenuated for MZ twin pairs, this would indicate the presence of genetic confounding (McGue 2010).

Furthermore, several sensitivity analyses were performed. First, we included SA more than 365 days in a separate analysis and conducted analyses with an overall measure of unemployment and SA without categorising them into different lengths. Then, a sensitivity analysis by excluding individuals younger than 19 years or older than 60 years was conducted as they were assumed to have a lower risk of unemployment, SA and DP. However, the results of main analyses were retained in the sensitivity analyses therefore we chose not to present these results. Furthermore, we tested a combined measure of LMM, which was defined as combined unemployment, SA and DP, in order to get a measure of the total burden of LMM. The combined measure of LMM yielded similar results, these are presented in Supplementary Table 1. All analyses were conducted by SAS Statistical Software version 9.4.

## Results

During the mean follow-up time of 8.7 years (SD 5.4), 17,118 individuals (30.2%) were unemployed, 27,783 individuals (48.9%) were granted SA benefits and 5979 individuals (10.5%) were granted DP. Young individuals, aged 16–25 years (74.0%), more commonly experienced a total burden of LMM compared with other age groups. Moreover, individuals in this young age group reported a lower score on job demands (4.60) and job control (5.87), but a higher score on social support (6.92) than those in older age groups (Table 1).

In the crude models, each one-unit increase in social support was significantly associated with an increased risk of unemployment < 365 days (range of HR 1.14–1.36), while each one-unit increase in social support was associated with a decrease in unemployment longer than one year during follow-up across the age groups (range of HR 0.77–0.91) (Table 2). On the other hand, each one-unit increase in job demands (range of HR 0.55–0.88) and job control (range of HR 0.80–0.95) predicted a decrease risk of unemployment in the crude models, regardless of unemployment length and age groups. Generally, these associations remained after further adjusting for sex, education, marital status, children living at home and type of living area. However, psychosocial working conditions had major influence on unemployment of 1–30 days among individuals older than 35 years and on unemployment > 365 days among individuals in age groups between 16 and 55 years in the multivariate models (Table 2).

**Table 2:** Cox proportional hazard ratios (HR) with 95% confidence intervals (CI) of psychosocial working conditions for being unemployed during the follow-up, stratified on age groups

		Crude model		Adjusted for sex		Adjusted for all covariates^a^		Co-twin analyses of discordant twin pairs								
								DZ^b^ same sex pairs + MZ^c^ pairs			DZ same sex pairs			MZ pairs		
	*n*	HR	95% CI	HR	95% CI	HR	95% CI	*n*	HR	95% CI	*n*	HR	95% CI	*n*	HR	95% CI
	*Unemployment 1–30 days*															
*Age 16–25 years*	387							141			55			86		
Job demands		0.88	0.75–1.04	0.89	0.76–1.05	0.89	0.75–1.05		0.98	0.75–1.27		0.87	0.58–1.30		1.06	0.75–1.50
Job control		0.93	0.86–1.01	0.98	0.90–1.06	0.97	0.89–1.05		1.00	0.87–1.14		0.98	0.79–1.22		1.01	0.84–1.20
Social support		**1.31**	**1.06–1.62**	1.12	0.89–1.40	1.12	0.90–1.41		1.22	0.85–1.74		1.65	0.92–2.94		0.96	0.61–1.53
*Age 26–35 years*	585							210			95			115		
Job demands		**0.74**	**0.66–0.83**	**0.74**	**0.66–0.83**	**0.83**	**0.73–0.95**		0.87	0.71–1.07		0.92	0.67–1.27		0.84	0.64–1.10
Job control		**0.88**	**0.82–0.93**	**0.90**	**0.85–0.96**	0.96	0.90–1.03		0.98	0.88–1.09		0.90	0.77–1.05		1.04	0.90–1.20
Social support		**1.36**	**1.17–1.59**	**1.22**	**1.02–1.45**	1.19	1.00–1.42		0.97	0.75–1.26		0.95	0.66–1.37		0.99	0.68–1.43
*Age 36–45 years*	495							171			111			60		
Job demands		**0.68**	**0.60–0.77**	**0.70**	**0.62–0.79**	**0.80**	**0.69–0.92**		0.97	0.77–1.24		0.98	0.73–1.30		0.98	0.63–1.51
Job control		**0.80**	**0.75–0.85**	**0.82**	**0.77–0.88**	**0.89**	**0.82–0.96**		0.97	0.85–1.10		0.93	0.79–1.09		1.05	0.84–1.30
Social support		**1.32**	**1.12–1.55**	1.07	0.88–1.30	1.03	0.85–1.25		1.13	0.86–1.48		1.15	0.81–1.61		1.09	0.70–1.71
*Age 46–55 years*	406							154			87			67		
Job demands		**0.67**	**0.58–0.77**	**0.67**	**0.58–0.77**	**0.80**	**0.68–0.94**		0.84	0.67–1.06		0.84	0.62–1.14		0.84	0.60–1.19
Job control		**0.86**	**0.80–0.93**	**0.84**	**0.78–0.90**	**0.91**	**0.84–0.99**		0.95	0.84–1.08		0.93	0.80–1.09		0.97	0.80–1.19
Social support		**1.18**	**1.01–1.39**	**1.41**	**1.15–1.73**	**1.31**	**1.07–1.61**		1.18	0.90–1.54		1.16	0.81–1.67		1.18	0.79–1.77
*Age 56–64 years*	164							47			24			23		
Job demands		**0.55**	**0.44–0.69**	**0.54**	**0.43–0.67**	**0.55**	**0.43–0.71**		0.86	0.57–1.29		0.85	0.52–1.39		0.87	0.41–1.83
Job control		0.96	0.85–1.07	0.93	0.82–1.06	1.01	0.87–1.16		1.14	0.91–1.44		0.97	0.70–1.34		1.37	0.96–1.96
Social support		**1.35**	**1.05–1.75**	**1.83**	**1.30–2.58**	**1.69**	**1.19–2.40**		1.13	0.66–1.92		1.29	0.64–2.61		0.94	0.42–2.12
	*Unemployment 31–365 days*															
*Age 16–25 years*	2153							475			206			269		
Job demands		**0.88**	**0.83–0.95**	**0.89**	**0.83–0.96**	**0.91**	**0.85–0.97**		0.92	0.80–1.06		**0.79**	**0.63–0.99**		1.03	0.85–1.24
Job control		**0.95**	**0.91–0.98**	0.97	0.93–1.00	0.97	0.94–1.01		0.99	0.92–1.06		1.03	0.92–1.15		0.96	0.87–1.05
Social support		1.08	0.99–1.18	1.00	0.91–1.10	1.00	0.91–1.10		0.93	0.77–1.13		0.90	0.69–1.17		0.97	0.74–1.28
*Age 26–35 years*	2557							774			349			425		
Job demands		**0.86**	**0.82–0.91**	**0.86**	**0.82–0.91**	**0.91**	**0.85–0.97**		0.99	0.89–1.09		0.94	0.81–1.09		1.03	0.90–1.18
Job control		**0.89**	**0.86–0.92**	**0.90**	**0.87–0.93**	**0.92**	**0.89–0.95**		0.96	0.91–1.02		0.96	0.89–1.05		0.96	0.90–1.03
Social support		**1.14**	**1.07–1.23**	**1.10**	**1.01–1.19**	1.08	0.99–1.17		1.08	0.95–1.22		1.11	0.91–1.36		1.05	0.90–1.24
*Age 36–45 years*	1921							634			358			276		
Job demands		**0.81**	**0.76–0.86**	**0.81**	**0.76–0.87**	**0.91**	**0.85–0.98**		0.98	0.87–1.10		1.00	0.86–1.16		0.95	0.81–1.13
Job control		**0.85**	**0.82–0.88**	**0.85**	**0.82–0.88**	**0.89**	**0.86–0.93**		**0.93**	**0.87–0.99**		**0.88**	**0.82–0.96**		0.99	0.90–1.09
Social support		1.07	0.99–1.16	1.01	0.92–1.11	0.99	0.90–1.09		0.96	0.84–1.11		1.04	0.86–1.26		0.87	0.71–1.08
*Age 46–55 years*	1420							509			317			192		
Job demands		**0.78**	**0.72–0.84**	**0.78**	**0.72–0.84**	0.93	0.85–1.01		0.92	0.82–1.05		0.90	0.77–1.05		0.96	0.79–1.18
Job control		**0.89**	**0.85–0.92**	**0.85**	**0.82–0.89**	**0.91**	**0.87–0.96**		0.93	0.87–0.99		0.92	0.84–1.00		0.96	0.86–1.07
Social support		0.94	0.87–1.02	1.03	0.93–1.15	0.97	0.88–1.07		1.00	0.86–1.16		1.05	0.88–1.25		0.91	0.70–1.18
*Age 56–64 years*	576							187			107			80		
Job demands		**0.80**	**0.71–0.90**	**0.78**	**0.69–0.88**	0.95	0.82–1.09		1.17	0.93–1.46		1.20	0.89–1.63		1.13	0.81–1.57
Job control		**0.89**	**0.84–0.95**	**0.82**	**0.77–0.88**	**0.89**	**0.83–0.96**		1.00	0.90–1.11		1.00	0.86–1.15		1.00	0.86–1.16
Social support		**0.84**	**0.75–0.95**	1.01	0.86–1.18	0.94	0.80–1.10		0.98	0.75–1.29		0.97	0.67–1.39		1.00	0.67–1.49
	*Unemployment > 365 days*															
*Age 16–25 years*	1006							222			105			117		
Job demands		**0.80**	**0.72–0.89**	**0.79**	**0.71–0.88**	**0.87**	**0.78–0.96**		0.97	0.77–1.22		0.84	0.61–1.16		1.11	0.81–1.54
Job control		**0.88**	**0.84–0.93**	**0.85**	**0.81–0.90**	**0.88**	**0.83–0.92**		0.91	0.82–1.01		**0.81**	**0.70–0.94**		1.01	0.87–1.16
Social support		**0.77**	**0.69–0.87**	**0.80**	**0.71–0.91**	**0.83**	**0.73–0.95**		0.98	0.75–1.29		1.07	0.73–1.58		0.88	0.60–1.30
*Age 26–35 years*	1665							454			226			228		
Job demands		**0.71**	**0.66–0.76**	**0.71**	**0.66–0.76**	**0.82**	**0.76–0.89**		0.91	0.80–1.04		0.88	0.72–1.06		0.93	0.78–1.12
Job control		**0.81**	**0.78–0.84**	**0.80**	**0.77–0.83**	**0.86**	**0.82–0.89**		0.93	0.87–0.99		0.96	0.86–1.06		0.90	0.82–1.00
Social support		1.05	0.97–1.15	1.03	0.93–1.13	0.98	0.89–1.08		1.05	0.89–1.24		1.16	0.92–1.46		0.95	0.76–1.20
*Age 36–45 years*	1534							431			265			166		
Job demands		**0.71**	**0.67–0.77**	**0.70**	**0.65–0.75**	**0.83**	**0.76–0.90**		0.82	0.71–0.94		**0.78**	**0.66–0.92**		0.90	0.70–1.16
Job control		**0.83**	**0.80–0.87**	**0.80**	**0.77–0.83**	**0.88**	**0.84–0.92**		0.92	0.85–0.99		**0.89**	**0.81–0.98**		0.96	0.86–1.08
Social support		**0.83**	**0.77–0.90**	**0.90**	**0.81–0.99**	**0.87**	**0.79–0.97**		1.01	0.87–1.18		0.94	0.77–1.14		1.14	0.89–1.46
*Age 46–55 years*	1604							538			343			195		
Job demands		**0.68**	**0.64–0.73**	**0.67**	**0.63–0.72**	**0.75**	**0.69–0.81**		**0.86**	**0.76–0.97**		0.88	0.75–1.03		0.82	0.67–1.01
Job control		**0.87**	**0.84–0.90**	**0.83**	**0.80–0.86**	**0.88**	**0.84–0.92**		**0.92**	**0.86–0.98**		0.94	0.87–1.01		**0.88**	**0.79–0.98**
Social support		**0.91**	**0.85–0.98**	1.05	0.95–1.15	0.99	0.90–1.08		0.98	0.86–1.12		1.01	0.86–1.19		0.91	0.72–1.17
*Age 56–64 years*	645							177			102			75		
Job demands		**0.79**	**0.70–0.88**	**0.78**	**0.69–0.87**	0.97	0.85–1.11		1.07	0.85–1.34		1.01	0.74–1.39		1.14	0.81–1.61
Job control		**0.92**	**0.87–0.97**	**0.88**	**0.83–0.94**	0.97	0.90–1.04		1.06	0.95–1.20		1.05	0.90–1.22		1.09	0.90–1.31
Social support		0.92	0.82–1.03	1.00	0.86–1.17	0.92	0.79–1.07		0.93	0.73–1.19		0.86	0.61–1.22		1.01	0.71–1.43

Statistically significant HR and 95% CI in boldface

^a^Adjusted for sex, education, marital status, children living at home, type of living area and sickness absence at baseline

^b^*DZ* dizygotic

^c^*MZ* monozygotic

Similarly, each one-unit increase in job demands and job control were significantly associated with a decrease risk of SA of 1–30 days in the multivariate models (range of HR 0.86–0.90). This association was also found in SA of 31–365 days, particularly for those older than 35 years (range of HR 0.87–0.93). Each one-unit increase in social support was associated with an increased risk of SA (regardless of lengths) in older individuals even after controlling for all covariates (range of HR 1.08–1.15) (Table 3).

**Table 3:** Cox proportional hazard ratios (HR) with 95% confidence intervals (CI) of psychosocial working conditions for being granted sickness benefits during the follow-up, stratified on age groups

	*n*	Crude model		Adjusted for sex		Adjusted for all covariates^a^		Co-twin analyses of discordant twin pairs								
								DZ^b^ same sex pairs + MZ^c^ pairs			DZ same sex pairs			MZ pairs		
		HR	95% CI	HR	95% CI	HR	95% CI	*n*	HR	95% CI	*n*	HR	95% CI	*n*	HR	95% CI
	*Sickness absence 1–30 days*															
*Age 16–25 years*	1589							438			165			273		
Job demands		**0.85**	**0.79–0.92**	**0.87**	**0.81–0.94**	**0.90**	**0.83–0.98**		0.95	0.82–1.11		0.92	0.72–1.17		0.98	0.81–1.18
Job control		0.96	0.92–1.00	1.02	0.98–1.06	1.03	0.99–1.08		1.06	0.98–1.15		**1.16**	**1.02–1.32**		1.01	0.92–1.11
Social support		**1.24**	**1.12–1.37**	1.00	0.90–1.12	1.02	0.91–1.14		1.05	0.87–1.28		1.01	0.76–1.35		1.09	0.84–1.40
*Age 26–35 years*	2986							851			401			450		
Job demands		**0.80**	**0.76–0.84**	**0.80**	**0.77–0.85**	**0.90**	**0.85–0.95**		0.97	0.87–1.07		0.97	0.84–1.12		0.97	0.84–1.11
Job control		**0.84**	**0.82–0.87**	**0.88**	**0.86–0.91**	**0.93**	**0.90–0.96**		0.95	0.90–1.00		0.96	0.89–1.04		0.94	0.88–1.01
Social support		**1.20**	**1.13–1.29**	0.94	0.88–1.02	**0.92**	**0.86–0.99**		0.97	0.86–1.09		1.03	0.86–1.24		0.92	0.80–1.07
*Age 36–45 years*	3138							914			519			395		
Job demands		**0.74**	**0.70–0.78**	**0.76**	**0.72–0.79**	**0.86**	**0.81–0.90**		0.87	0.79–0.96		**0.82**	**0.73–0.93**		0.95	0.81–1.10
Job control		**0.83**	**0.81–0.85**	**0.86**	**0.84–0.89**	**0.92**	**0.89–0.95**		0.94	0.89–0.99		**0.92**	**0.86–0.99**		0.97	0.89–1.05
Social support		**1.28**	**1.20–1.36**	1.02	0.95–1.11	0.98	0.91–1.06		1.05	0.94–1.18		1.06	0.92–1.22		1.05	0.88–1.26
*Age 46–55 years*	3239							1051			607			444		
Job demands		**0.82**	**0.78–0.86**	**0.83**	**0.79–0.87**	**0.89**	**0.84–0.95**		0.91	0.83–1.00		**0.88**	**0.78–0.99**		0.95	0.84–1.08
Job control		**0.87**	**0.85–0.90**	**0.89**	**0.87–0.92**	**0.93**	**0.90–0.95**		0.99	0.95–1.04		0.97	0.92–1.04		1.02	0.94–1.10
Social support		**1.25**	**1.19–1.33**	**1.13**	**1.06–1.22**	**1.08**	**1.01–1.16**		1.11	1.01–1.23		1.13	0.99–1.28		1.09	0.93–1.28
*Age 56–64 years*	1212							382			217			165		
Job demands		**0.80**	**0.74–0.87**	**0.82**	**0.75–0.88**	**0.90**	**0.82–0.99**		1.01	0.87–1.17		0.92	0.75–1.12		1.15	0.91–1.43
Job control		**0.88**	**0.85–0.92**	**0.91**	**0.87–0.95**	0.96	0.91–1.00		0.94	0.87–1.02		0.94	0.84–1.05		0.95	0.84–1.06
Social support		**1.31**	**1.19–1.43**	**1.21**	**1.07–1.37**	**1.15**	**1.01–1.29**		1.13	0.94–1.36		1.00	0.78–1.27		**1.35**	**1.01–1.79**
	*Sickness absence 31–365 days*															
*Age 16–25 years*	1823							464			182			282		
Job demands		**0.89**	**0.83–0.96**	**0.91**	**0.85–0.98**	0.95	0.88–1.03		0.96	0.84–1.11		0.93	0.73–1.18		0.98	0.82–1.17
Job control		**0.92**	**0.89–0.95**	0.98	0.95–1.02	1.00	0.96–1.04		0.98	0.92–1.06		0.97	0.86–1.09		1.00	0.91–1.09
Social support		**1.12**	**1.02–1.23**	0.88	0.80–0.97	0.90	0.82–1.00		0.97	0.80–1.18		0.95	0.71–1.28		0.99	0.77–1.26
*Age 26–35 years*	3502							916			430			486		
Job demands		**0.87**	**0.83–0.91**	**0.88**	**0.84–0.92**	0.97	0.92–1.02		0.99	0.90–1.08		0.99	0.87–1.14		0.98	0.86–1.12
Job control		**0.88**	**0.86–0.91**	**0.93**	**0.91–0.96**	0.98	0.95–1.00		0.96	0.91–1.01		0.93	0.86–1.00		0.99	0.92–1.06
Social support		**1.34**	**1.26–1.42**	1.07	1.00–1.15	1.06	0.99–1.13		1.11	0.99–1.25		1.20	1.00–1.44		1.05	0.90–1.22
*Age 36–45 years*	4012							1076			646			430		
Job demands		**0.85**	**0.81–0.89**	**0.86**	**0.82–0.90**	**0.93**	**0.88–0.98**		0.99	0.91–1.08		1.03	0.92–1.15		0.94	0.82–1.08
Job control		**0.89**	**0.87–0.91**	**0.91**	**0.89–0.94**	**0.96**	**0.93–0.98**		0.98	0.93–1.03		0.98	0.91–1.04		0.97	0.90–1.05
Social support		**1.23**	**1.16–1.30**	**1.10**	**1.03–1.17**	**1.09**	**1.02–1.16**		1.01	0.91–1.13		1.00	0.87–1.14		1.04	0.87–1.23
*Age 46–55 years*	4415							1282			768			514		
Job demands		**0.85**	**0.82–0.89**	**0.85**	**0.82–0.89**	**0.92**	**0.88–0.97**		0.94	0.87–1.02		0.90	0.81–1.00		1.01	0.89–1.14
Job control		**0.90**	**0.88–0.93**	**0.92**	**0.90–0.94**	**0.96**	**0.93–0.98**		0.95	0.91–0.99		**0.93**	**0.88–0.98**		0.97	0.91–1.04
Social support		**1.21**	**1.16–1.27**	**1.14**	**1.08–1.21**	**1.09**	**1.03–1.16**		1.10	1.01–1.20		1.07	0.96–1.20		1.14	0.99–1.31
*Age 56–64 years*	1831							534			311			223		
Job demands		**0.84**	**0.79–0.90**	**0.85**	**0.79–0.90**	**0.87**	**0.81–0.94**		0.90	0.79–1.01		0.91	0.77–1.06		0.89	0.73–1.07
Job control		**0.90**	**0.87–0.93**	**0.92**	**0.88–0.95**	**0.94**	**0.90–0.98**		0.97	0.91–1.03		0.95	0.87–1.04		0.99	0.90–1.09
Social support		**1.19**	**1.10–1.28**	**1.12**	**1.02–1.23**	1.09	0.99–1.21		0.99	0.87–1.12		1.06	0.89–1.26		0.91	0.75–1.11

Statistically significant HR and 95% CI in boldface

^a^Adjusted for sex, education, marital status, children living at home, type of living area and sickness absence at baseline

^b^*DZ* dizygotic

^*c*^*MZ* monozygotic

Regarding DP, each one-unit increase in social support predicted an increased risk in all age groups except for the youngest age group in the crude models (range of HR 1.32–1.54) (Table 4). This association remained only for individuals between 56 and 64 years in fully adjusted models (HR 1.16, 95% CI 1.04–1.30). Furthermore, the significant associations between increased job demands and job control with lower risk of DP were found in the multivariate models, primarily among individuals older than 45 years (range of HR 0.86–0.94) (Table 4).

**Table 4:** Cox proportional hazard ratios (HR) with 95% confidence intervals (CI) of psychosocial working conditions for being granted disability pension* during the follow-up, stratified on age groups

	*n*	Crude model		Adjusted for sex		Adjusted for all covariates^a^		Co-twin analyses of discordant twin pairs								
								DZ^b^ same sex pairs + MZ^c^ pairs			DZ same sex pairs			MZ pairs		
		HR	95% CI	HR	95% CI	HR	95% CI	n	HR	95% CI	n	HR	95% CI	n	HR	95% CI
*Age 16–25 years*	249							67			25			42		
Job demands		**0.73**	**0.60–0.90**	**0.76**	**0.62–0.92**	**0.80**	**0.66–0.98**		0.94	0.64–1.36		0.84	0.47–1.51		1.02	0.62–1.67
Job control		0.90	0.82–1.00	0.97	0.87–1.07	0.99	0.89–1.09		0.94	0.79–1.12		0.94	0.71–1.25		0.94	0.75–1.18
Social support		1.20	0.93–1.56	0.93	0.71–1.22	0.97	0.74–1.27		0.75	0.40–1.39		0.61	0.25–1.46		0.89	0.36–2.23
*Age 26–35 years*	770							223			116			107		
Job demands		**0.74**	**0.67–0.82**	**0.76**	**0.69–0.83**	0.95	0.85–1.06		0.99	0.83–1.19		0.93	0.72–1.19		1.07	0.83–1.39
Job control		**0.83**	**0.79–0.88**	**0.89**	**0.85–0.94**	1.00	0.94–1.06		0.95	0.86–1.05		0.93	0.81–1.07		0.97	0.84–1.12
Social support		**1.34**	**1.18–1.53**	0.95	0.82–1.10	0.92	0.79–1.07		1.11	0.88–1.40		0.98	0.71–1.37		1.24	0.89–1.75
*Age 36–45 years*	1334							399			236			163		
Job demands		**0.78**	**0.72–0.84**	**0.81**	**0.75–0.87**	0.97	0.89–1.06		0.97	0.84–1.12		1.00	0.83–1.21		0.92	0.75–1.16
Job control		**0.83**	**0.80–0.86**	**0.89**	**0.85–0.93**	0.98	0.93–1.03		1.01	0.93–1.10		1.04	0.93–1.16		0.98	0.86–1.11
Social support		**1.54**	**1.39–1.70**	1.08	0.95–1.21	1.04	0.93–1.17		0.99	0.85–1.16		0.99	0.80–1.21		1.00	0.78–1.27
*Age 46–55 years*	2156							639			412			227		
Job demands		**0.74**	**0.70–0.79**	**0.75**	**0.71–0.80**	**0.86**	**0.80–0.92**		0.96	0.86–1.08		0.93	0.80–1.08		1.03	0.85–1.25
Job control		**0.84**	**0.81–0.87**	**0.88**	**0.85–0.91**	**0.94**	**0.91–0.98**		0.96	0.90–1.02		0.94	0.87–1.01		1.01	0.91–1.13
Social support		**1.37**	**1.27–1.47**	**1.13**	**1.04–1.23**	1.05	0.96–1.14		0.98	0.86–1.11		0.98	0.84–1.14		0.98	0.79–1.21
*Age 56–64 years*	1470							394			229			165		
Job demands		**0.75**	**0.70–0.81**	**0.77**	**0.71–0.82**	**0.87**	**0.80–0.95**		0.92	0.80–1.06		0.95	0.78–1.15		0.89	0.71–1.11
Job control		**0.84**	**0.81–0.86**	**0.86**	**0.83–0.90**	**0.92**	**0.88–0.96**		0.94	0.86–1.01		0.93	0.84–1.04		0.94	0.84–1.07
Social support		**1.32**	**1.22–1.44**	**1.23**	**1.11–1.38**	**1.16**	**1.04–1.30**		1.06	0.89–1.26		1.15	0.91–1.44		0.95	0.74–1.23

Statistically significant HR and 95% CI in boldface

*Including disability pension and sickness absence > 365 days

^a^Adjusted for sex, education, marital status, children living at home, type of living area and sickness absence at baseline

^b^*DZ* dizygotic

^c^*MZ* monozygotic

In the discordant twin pair analyses, the HRs did change slightly and became non-significant across all age groups (Tables 2, 3, 4).

## Discussion

This study of a large population-based cohort of 56,867 twins showed that psychosocial working conditions are potential risk factors for LMM, i.e., unemployment, SA and DP. Specifically, we found that each one-unit increase in job demands or control were predictors for a lower risk of unemployment, SA and DP. On the other hand, each one-unit increase in social support was associated with an increased risk of unemployment of between 1 and 30 days among individuals older than 45 years while an inverse relationship was found for unemployment longer than 365 days in individuals aged between 16 and 45 years. Social support was also a risk factor for SA and DP. However, we found that familial factors seem to influence nearly all the associations.

According to the Demand–Control–Support model proposed by Karasek and Theorell, jobs with high demands, low control and low social support are associated with adverse health and LMM (Karasek 1979). In this study, we examined the separate role of job demands, job control and social support in relation with LMM and in agreement with the previous research, our findings showed that each one-unit increase of job control generally was associated with a lower risk of unemployment and short-term SA across age groups (Norberg et al. 2020; Farrants et al. 2020). The same impact from job control was also found mainly in older individuals for long-term SA and DP. This result points to the importance of psychosocial influence at work for future labour market exit. Conversely, we found an association between each one-unit increase of job demands and lower risk of LMM, which is not in line with the theory. In spite of this, several studies have shown that low job demands and low job control represent passive workers and those workers often experience a decline in physical activity and problem-solving skills as well as an increase in depression and hopelessness (Karasek 1979; Hellerstedt 1997). This might be an explanation for the results of associations with future unemployment and work incapacity. In addition, our divergent results might be due to the use of a JEM, while quite a few of the previous studies applied self-reported survey data (Mather 2015; Canivet 2013; Lahelma 2012; Clumeck 2009). Several studies which applied JEM for measuring psychosocial working conditions, reported a high risk of SA and DP in occupations with low demands and control (Samuelsson 2013; Norberg et al. 2020; Ropponen 2013), which was confirmed by our study. However, future studies with separate measure of job demands, job control and social support are warranted to replicate our results.

The literature in regard to the association between low social support and LMM shows inconsistent results. For example, Carr et al. (2016) found no association between low social support and work exit. In contrast, Fleischmann et al. (2018) and Lund et al. (2005) found that high social support in middle-aged and older workers decreased the risk of LMM. In this study, we further investigated this association and found that social support decreased the risk of long-term unemployment, particularly among young and middle-aged individuals. Nevertheless, since this is among the first studies to focus on several age groups and duration of unemployment, more studies are needed to confirm the current findings.

In addition, although diverse results were detected in different age groups in fully adjusted models, the HRs were around the same magnitude and direction across the age groups. This finding might imply that the impact of psychosocial working conditions on LMM is generally similar for all ages. Previous research has mainly focused on general or specific age groups while exploring the association between psychosocial working conditions and LMM in different age groups, as in the present study, shed additional light on the association from a life course perspective.

In this study, we also used a twin study design, which provides the benefit of controlling for many unmeasured confounders in terms of genetics and early shared environment. We observed that the estimates changed slightly. Still, almost all significant results of the whole cohort regarding the associations between psychosocial working conditions and various measures of LMM disappeared in discordant twin pair analyses. This supports the assumption that familial confounding cannot be ruled out, i.e., genetics and shared (mainly childhood) environment may play a role in the associations. However, the results need to be replicated, preferably with even larger sample sizes, to draw firm conclusion regarding familial confounding. However, one potential pathway might be that poor psychosocial working conditions are associated with mental or physical ill-health due to genetic susceptibility, which may affect subsequent LMM. Alternatively, personality or socioeconomic status which are known to be affected by genetics may affect the associations with LMM (Sanchez-Roige 2018; Torvik 2015).

## Strengths and limitations

The main strength of this study includes the large sample size (*n* = 56,867), the long follow-up (mean 8.7 years), a prospective cohort design and the use of Swedish nationwide register data of high quality (Dödsorsaksregistret 2015; Ludvigsson 2011), which reduced the risk of loss to follow-up and recall bias. A further strength is including discordant twin pairs in the analyses, which enable to control for the influence from familial factors. However, in some co-twin analysis, we might have lacked power due to few identified discordant twin pairs in stratified groups. Thus, the results in those groups should be interpreted with caution. We were also able to take several relevant covariates into account in relation to the exposures and outcomes. Still, there might be other relevant factors we could not include as covariates, such as unhealthy lifestyle (i.e., smoking behaviour, alcohol consumption and lack of physical activity) or organisational information. Using a validated JEM, we could obtain more comprehensive information on job demands, job control and social support and eliminate reporting bias in relation to health (Kolstad 2011; Rugulies 2012; Petersen 2020). However, we lacked detailed values for all occupations and information on occupation was only available at baseline and not updated in LISA for all individuals every year. Another limitation includes that data on sick-leave spells < 14 days were not available for employed individuals. Thus, the findings from the study may not be generalisable to individuals with short-term SA spells (< 14 days). Still, the findings in this study can be generalised to working-aged individuals living in countries with comparable economic and labour market situations and health care and social insurance systems. Further, twins are similar to the population at large for most exposures and outcomes including SA and DP (Narusyte 2011).

## Conclusions

This population-based prospective twin study showed that job demands, job control and social support were risk factors for LMM (i.e., unemployment, SA and DP). The risk of unemployment, SA and DP was associated with low job demands and low job control while high social support decreased the risk of long-term unemployment, particularly among young and middle-aged individuals. Hence, to facilitate a sustainable working life one strategy might be improving psychosocial working conditions (e.g., promote high job control and high social support at workplaces), which is likely to be applicable to all age groups and has effect on both short-term and long-term unemployment and SA. Awareness of these factors at workplace or occupational health care for prevention of LMM is recommended. Moreover, familial factors may play a role in explaining the associations between psychosocial working conditions and LMM.

## Supplementary Information

Below is the link to the electronic supplementary material.

Supplementary file1 (PDF 200 kb)

## Data Availability

The data that support the findings of this study are available from the original sources: the Swedish Twin Registry, Statistics Sweden, Swedish Social Insurance Agency and The Swedish National Board of Health and Welfare. Restrictions apply to the availability of the data used in this study based on the Swedish Twin project Of Disability pension and Sickness absence (STODS), which were used with ethical permission for the current study and therefore are not publicly available.
