# Supplementary material for: Effects of age on psychosocial working conditions and future labour market marginalisation: a cohort study of 56,867 Swedish twins
Source: Int Arch Occup Environ Health. 2021 May 7;95(1):199–211. doi: 10.1007/s00420-021-01704-z (PMC8755677; doi:10.1007/s00420-021-01704-z)
Supplement: Supplementary file 1 — Supplementary file1 (PDF 200 kb) [file 420_2021_1704_MOESM1_ESM.pdf]

Supplementary table

Table 1. Cox proportional hazard ratios (HR) with 95% confidence intervals (CI) of psychosocial working conditions for being labour market marginalised during the follow-up, stratified on age groups.

	Crude model		Adjusted for sex		Adjusted for all covariates[1]		Co-twin analyses of discordant twin pairs			
							DZ[2] same sex		MZ[3]	
	HR	95% CI	HR	95% CI	HR	95% CI	HR	95% CI	HR	95% CI
**Age 16-25 years**										
Job demands	**0.81**	**0.78-0.85**	**0.82**	**0.79-0.86**	**0.86**	**0.82-0.90**	0.81	0.62-1.06	0.92	0.74-1.15
Job control	**0.92**	**0.90-0.94**	**0.96**	**0.93-0.98**	0.97	0.95-1.00	0.99	0.88-1.12	0.97	0.87-1.08
Social support	**1.09**	**1.03-1.15**	0.94	0.89-1.00	0.96	0.91-1.02	1.16	0.84-1.60	1.07	0.79-1.44
**Age 26-35 years**										
Job demands	**0.79**	**0.77-0.82**	**0.79**	**0.77-0.82**	**0.89**	**0.86-0.92**	0.96	0.83-1.10	1.00	0.88-1.14
Job control	**0.83**	**0.82-0.85**	**0.87**	**0.86-0.88**	**0.92**	**0.90-0.93**	**0.91**	**0.85-0.97**	**0.93**	**0.86-0.99**
Social support	**1.30**	**1.25-1.36**	**1.05**	**1.01-1.10**	1.03	0.99-1.08	1.13	0.96-1.34	0.95	0.81-1.10
**Age 36-45 years**										
Job demands	**0.74**	**0.72-0.76**	**0.75**	**0.73-0.77**	**0.85**	**0.83-0.88**	**0.88**	**0.79-0.99**	0.94	0.82-1.08
Job control	**0.82**	**0.81-0.83**	**0.84**	**0.83-0.86**	**0.90**	**0.88-0.92**	**0.90**	**0.84-0.96**	0.99	0.91-1.08
Social support	**1.24**	**1.20-1.29**	1.04	0.99-1.09	1.01	0.96-1.05	1.03	0.90-1.17	0.95	0.80-1.13
**Age 46-55 years**										
Job demands	**0.75**	**0.73-0.77**	**0.76**	**0.74-0.78**	**0.85**	**0.83-0.88**	**0.81**	**0.73-0.90**	0.95	0.84-1.08
Job control	**0.85**	**0.83-0.86**	**0.86**	**0.85-0.88**	**0.91**	**0.90-0.93**	**0.91**	**0.86-0.97**	0.99	0.92-1.06
Social support	**1.25**	**1.21-1.29**	**1.16**	**1.11-1.20**	**1.08**	**1.04-1.13**	1.06	0.95-1.19	1.04	0.89-1.21
**Age 56-64 years**										
Job demands	**0.77**	**0.74-0.80**	**0.78**	**0.75-0.81**	**0.88**	**0.84-0.92**	**0.85**	**0.74-0.99**	0.87	0.73-1.04
Job control	**0.87**	**0.86-0.89**	**0.88**	**0.86-0.90**	**0.94**	**0.91-0.96**	**0.92**	**0.85-0.99**	0.99	0.91-1.08
Social support	**1.22**	**1.17-1.28**	**1.19**	**1.12-1.26**	**1.12**	**1.06-1.19**	1.07	0.92-1.25	1.01	0.83-1.21

[1] Adjusted for sex, education, marital status, children living at home, type of living area and sickness absence at baseline.
[2] DZ= dizygotic.
[3] MZ=monozygotic
Statistically significant HR and 95% CI in boldface.
